# TCRvβ8 chimeric antigen receptor natural killer cells exhibit potent preclinical activity against T‐cell malignancies

**DOI:** 10.1002/ctm2.70004

**Published:** 2024-08-23

**Authors:** Lianjun He, Yinmei He, Ye He, Xing Bao, Yuqiong Yang, Xueyi Qian, Ziyun Lin, Weijie He, Yao Wu, Huimin Shao, Lingjie Zhou, Lin Wan, Zhenyu Xu

**Affiliations:** ^1^ Precision Medicine Centre The First Affiliated Hospital of Wannan Medical College (Yijishan Hospital of Wannan Medical College) Wuhu China; ^2^ Department of Pediatrics The First Affiliated Hospital of Shandong First Medical Medical University & Shandong Provincial Qianfoshan Hospital Jinan China; ^3^ Department of Radiotherapy The Second People's Hospital of Wuhu City Wuhu China; ^4^ Department of Hematology The First Affiliated Hospital of Wannan Medical College (Yijishan Hospital of Wannan Medical College) Wuhu China; ^5^ Department of Pharmacy The Second Affiliated Hospital of Wannan Medical College Wuhu China; ^6^ School of Pharmacy Wannan Medical College Wuhu China

Dear Editor

T‐cell malignancies, such as mainly T‐cell lymphomas and T‐cell acute lymphoblastic leukaemia (T‐ALL),^1^ are often associated with poor prognosis.^2^ The effectiveness of immunotherapy for treating T cell leukaemias was not promising.^3^ Recent research on therapeutic targets against T‐cell malignancies has primarily focused on CD5 or CD7.^4^, ^5^ However, targeting these T‐cell antigens has led to the occurrence of T‐cell disorders due to immune impairment. To address the above problems, we developed a chimeric antigen receptor‐natural killer (CAR‐NK) platform specifically eliminating malignant TCRvβ8 T‐cells while preserving the majority of normal T‐cells to avoid immune dysfunction. Both normal and malignant T‐cells express a unique TCR β chain,^6^ and clonal expansions of one or more TCRs are often observed in cases of T‐cell malignancies,^7^ making the TCR β chain an effective target for CAR therapy. While, there have been multiple reports about CAR‐T therapy for targeting TCRvβ,^8^, ^9^ using NK cells instead of autologous T‐cells for CAR‐T preparation, not only avoids the risk of contamination by malignant cells in the final product but also prevents fratricide during CAR‐T preparation. Additionally, NK cells from healthy individuals have higher vitality and safety.

We utilized a lentiviral system to construct four TCRvβ8 CAR‐NKs (Figure 1A). Among them, 4‐1BB‐CD3ζ exhibited higher transduction efficiency (Figure 1B and Figure S1A) and greater levels of cytotoxicity (Figure 1E), while showing no significant difference in terms of NK proportion (Figure 1C and Figure S1B) and expansion fold (Figure 1D). Therefore, we selected 4‐1BB‐CD3ζ CAR for further study.

**FIGURE 1:**
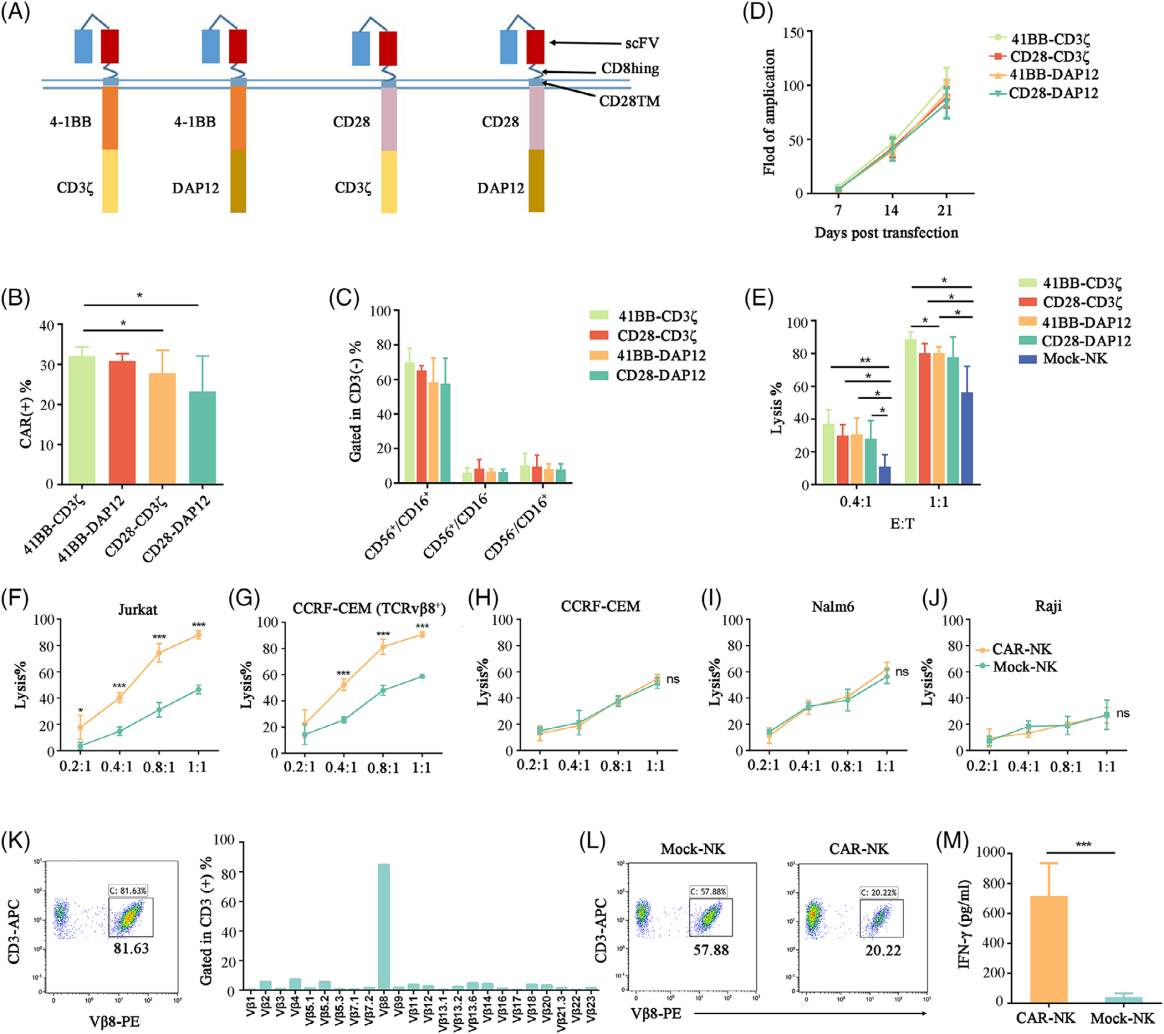
4‐1BB/CD3ζ chimeric antigen receptor‐natural killer (CAR‐NK) cells exhibited specific cytotoxicity against target cells in vitro. (A) Schema of four CARs which are designed to bind TCRvβ8 through extracellular scFV. (B) Transduction efficiency of above CARs in NK cells (*n* = 3, mean ± s.d.). (C) Evaluation of the impact of CARs structure on the expression of CD56^+^CD16^+^ in CAR‐NK cells (*n* = 3, mean ± s.d.). (D) The amplification fold change of four CAR‐NK cells (*n* = 3, mean ± s.d.). (E) The lysis percentage of Jurkat cells after cocultured with CAR‐NK or mock NK cells at effector‐to‐target ratios of 0.4:1 and 1:1 for 8 h (*n* = 3, mean ± s.d.). The lysis percentage of (F) Jurkat cells, (G) CCRF‐CEM (TCRvβ8^+^) cells, (H) CCRF‐CEM cells, (I) Nalm6 cells and (J) Raji cells, after cocultured with CAR‐NK or mock NK cells under different effector‐to‐target ratios for 8 h (*n* = 3, mean ± s.d.). (K) Malignant T‐cells derived from patients displayed a dominant Vβ8 clone by flow cytometry. (L) Remained percentage of TCRvβ8^+^ T‐cells in T‐cell malignancies after cocultured with CAR‐NK or mock NK cells with an effector‐to‐target ratio of 0.5:1 for 8 h. (M) The expression levels of interferon‐gamma (IFN‐γ) in cell supernatant of CAR‐NK or mock NK cells after cocultured with malignant T‐cells with an effector‐to‐target ratio of 0.5:1 for 8 h.

The phenotype of CAR‐NKs and Mock‐NKs is similar (Figure S2A), while an increase in CD107a and interferon‐gamma (IFN‐γ) expression on CAR‐NKs was induced after co‐culture with Jurkat cells (Figure S2B). CAR‐NKs exhibited promising cytotoxicity against TCRvβ8 positive cells (Figure 1F,G and Figure S3A,B) while having no killing effect on TCRvβ8^−^ cells (Figure 1H,J and Figure S3C–E). Furthermore, to assess the activity of CAR‐NKs against malignant T cells from a lymphoma patient, peripheral blood was collected and it was observed that CD3(+)/TCRvβ8(+) positive cells accounted for up to 80% by flow cytometry (Figure 1K). CAR‐NKs exhibited an enhanced ability to eliminate malignant T cells compared to Mock‐NKs (Figure 1L,M). Taken together, Vβ8‐CAR‐NKs may specifically target Vβ8^+^ T leukaemia cells in vitro.

To monitor CAR‐NK expansion and persistence, a repeated antigen stimulation protocol using Jurkat cells was developed (Figure S4A). TCRvβ8 CAR‐NKs got an enrichment of CAR^+^ cells and a continuous amplification after antigen stimulation (Figure S4B,C). Furthermore, CAR‐NKs maintained highly effective anti‐tumour activity after three rounds of stimulation (Figure S4D). In vivo, CAR‐NKs showed enrichment of CAR^+^ cells 1−2 weeks after injection, followed by subsequent loss of CAR expression (Figure S4E).

Because normal T‐cells are polyclonal, removing a portion of TCRvβ8^+^ T‐cells may not change the integrity of the total T‐cell repertoire. To confirm this hypothesis, we cocultured CAR‐NKs with five normal adult T‐cells. Indeed, CAR‐NKs eliminated TCRvβ8^+^ cells (Figure  2A,C) and had no difference in total TCRαβ^+^ expression compared with control NK groups (Figure 2A,B). Similarly, TCRvβ8 was significantly decreased in the CAR‐NK group, but the other TCRvβs were not significantly changed by flow cytometry and TCR sequencing analysis (Figure 2D and Figure S5A,B). To determine whether the loss of a T‐cell subtype would affect an immune response, three healthy donor peripheral blood TCRvβ8 T‐cells were removed by magnetic bead separation, and were exposed to viral peptides. Across all donors, there was no significant difference in the secretion of IFN‐γ between the sorting group and the control group (Figure 2E). These data prove designing CARs based on the malignant clones of each patient is an effective strategy for clearing T‐cell tumours.

**FIGURE 2:**
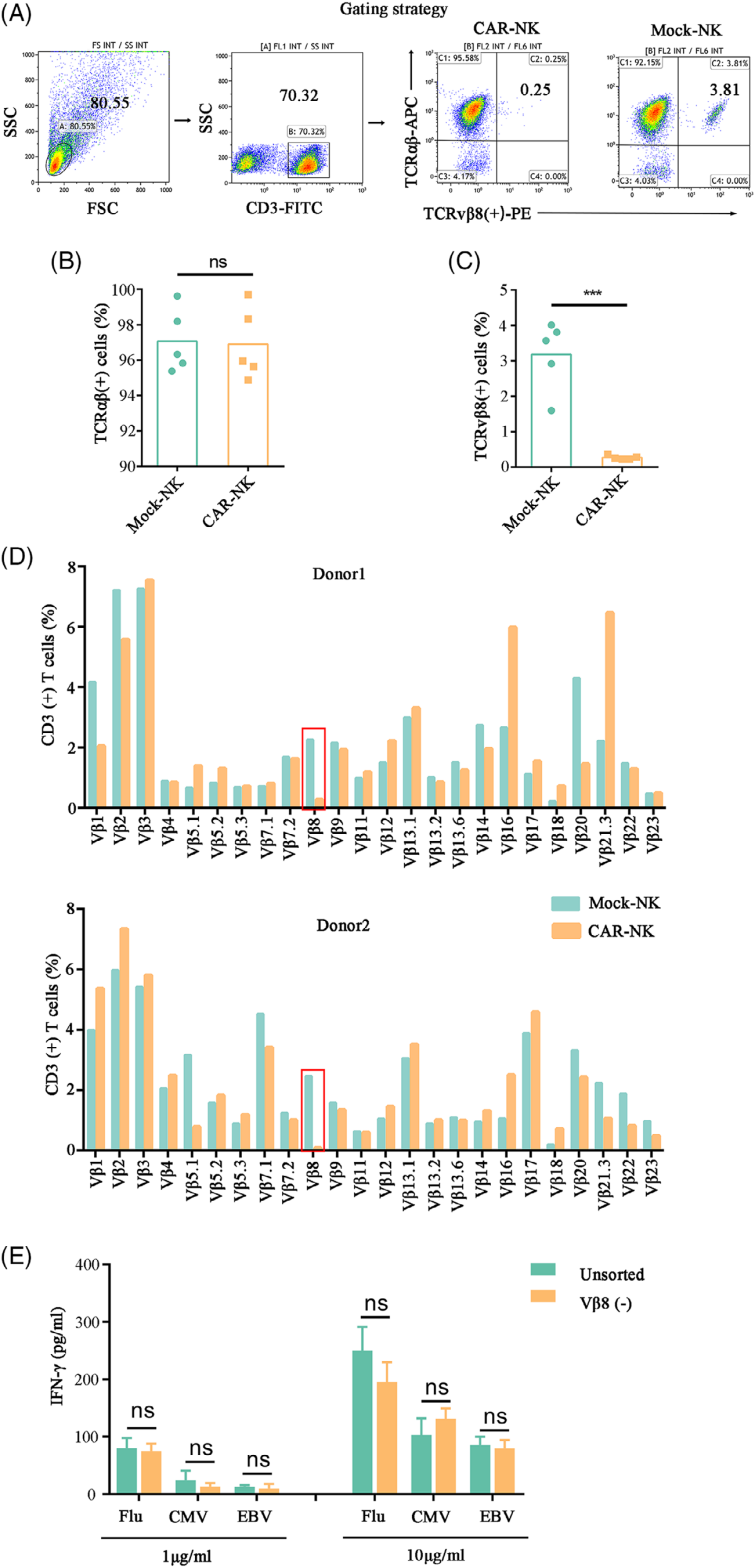
TCRvβ8^+^ chimeric antigen receptor‐natural killer (CAR‐NK) cells specifically eliminated TCRvβ8^+^ normal T cells while leaving the phenotype and activity of remaining normal T cells unaffected. (A, B) The percentage of TCRαβ^+^ normal T cells after cocultured with CAR‐NK or mock NK cells with an effector‐to‐target ratio of 0.5:1 for 24 h (*n* = 5, mean ± s.d.). (A–C) The percentage of TCRvβ8^+^ normal T cells after cocultured with CAR‐NK or mock NK cells with an effector‐to‐target ratio of 0.5:1 for 24 h (*n* = 5, mean ± s.d.). (D) TCRvβ expression in normal T cells after coculture with CAR‐NK or mock NK cells for 24 h by flow cytometry. (E) Followed by being stimulated with peptides, including Flu, CMV and EBV viruses, the expression level of interferon‐gamma (IFN‐γ) in the supernatant of normal T cells with or without being sorted to remove TCR Vβ8 population (*n* = 3, mean ± s.d.).

To identify the potential anti‐tumour effect of CAR may lead to dominant amplification of specific transcriptome subsets. We performed scRNA‐seq analyses on CAR^+^ and CAR^−^ NKs cocultured with Jurkat cells. Nineteen cell clusters were identified (Figure S6A,B). DEGs results showed that genes involving cell proliferation, DNA repair, cytotoxicity, and major metabolic pathways were significantly upregulated in CAR^+^ NKs, while genes involving cell cycle blockade were upregulated in CAR^−^ NKs (Figure S6C). Gene Ontology enrichment analyses revealed genes involved in oxidative phosphorylation and immunological synapse were upregulated in CAR^+^ NKs (Figure S6D). Specifically, cluster 5,12,14, exhibited high levels of cell proliferation and cytotoxicity signature genes, while cluster 17 exhibited high levels of maturation signature (Figure S6E–G). Taken together, these data suggested that CAR involvement in the killing of target cells may lead to differentiation and proliferation of effector cells.

Previous studies have suggested that antigen density may be a key factor in the primary and/or acquired resistance associated with CAR therapeutics.^10^ To investigate the influence of antigen density on the activity of CAR‐NKs, we used a lentiviral system to overexpress TCRvβ8 on CCRF‐CEM cells and established libraries expressing different densities of surface TCRvβ8 by flow cytometry (Figure 3A,B). CAR‐NKs demonstrated reduced killing capacity in response to cell lines expressing low levels of TCRvβ8 compared with those expressing high levels and the sensitivity of CAR‐NKs increased with the augmented proportion of effector cells (Figure 3C). In conclusion, the clinical efficacy of this CAR‐NK product can be predicted based on the antigen expression of the initial malignant cells.

**FIGURE 3:**
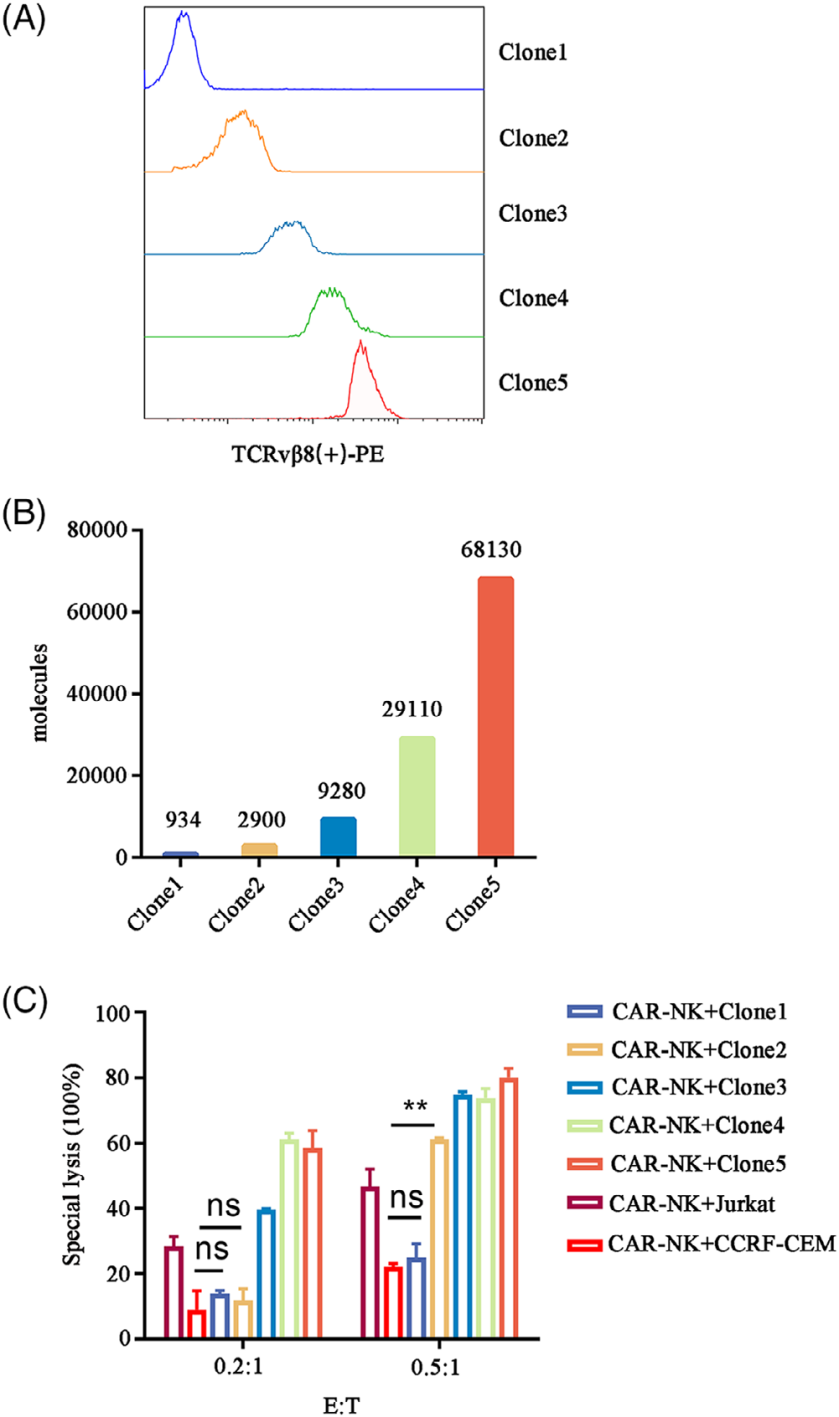
TCRvβ^+^ chimeric antigen receptor‐natural killer (CAR‐NK) cells exhibited potent activity against malignant T cells with high expression levels of TCRvβ8. (A) The expression level of TCRvβ8 on different groups of CCRF‐CEM (TCRvβ8^+^) cells. (B) The number of TCRvβ8 molecules in each group of CCRF‐CEM (TCRvβ8^+^) cells was determined by the BD Quantibrite Kit. (C) The lysis percentage of target cells after cocultured with CAR‐NK cells at an effector‐to‐target ratio of 0.2:1 and 0.5:1 after 8 h (*n* = 3, mean ± s.d.).

To determine whether CAR‐NKs have antitumor effects in vivo, we used NTG mice to establish a tumour‐bearing model (Figure 4A). It was found that the tumours in the CAR‐NKs group were significantly decreased compared to the control NKs group (Figure 4B,C). The survival period of the mice in the CAR‐NKs group was extended compared to that of the control NKs group (Figure 4D). Furthermore, CAR‐NKs were still detectable even 90 days post‐treatment (Figure 4E), while tumour cells were nearly undetectable (Figure 4F), indicating the persistent antitumor effect exerted by CAR‐NKs. Notably, CAR‐NKs didn't attack normal tissues and caused severe side effects after 2 weeks of injection (Figure S7). Subsequently, a xenograft model was established using patient‐derived T‐cell lymphoma cells in NTG mice (Figure 4G). Malignant T‐cells were significantly decreased after CAR‐NKs therapy, while the control NKs group displayed slow tumour progression, and the PBS group demonstrated rapid tumour burden progression (Figure 4H). As expected, the mice treated with CAR‐NKs exhibited significantly prolonged survival (Figure 4I). On day 47, malignant T‐cells were continuously inhibited (Figure 4J), and the presence of CAR‐NK cells was detected in the bone marrow, peripheral blood and spleen (Figure 4K). These findings indicated that CAR‐NKs possess persistent antitumor cell activity in vivo without causing harmful damage to normal tissues.

**FIGURE 4:**
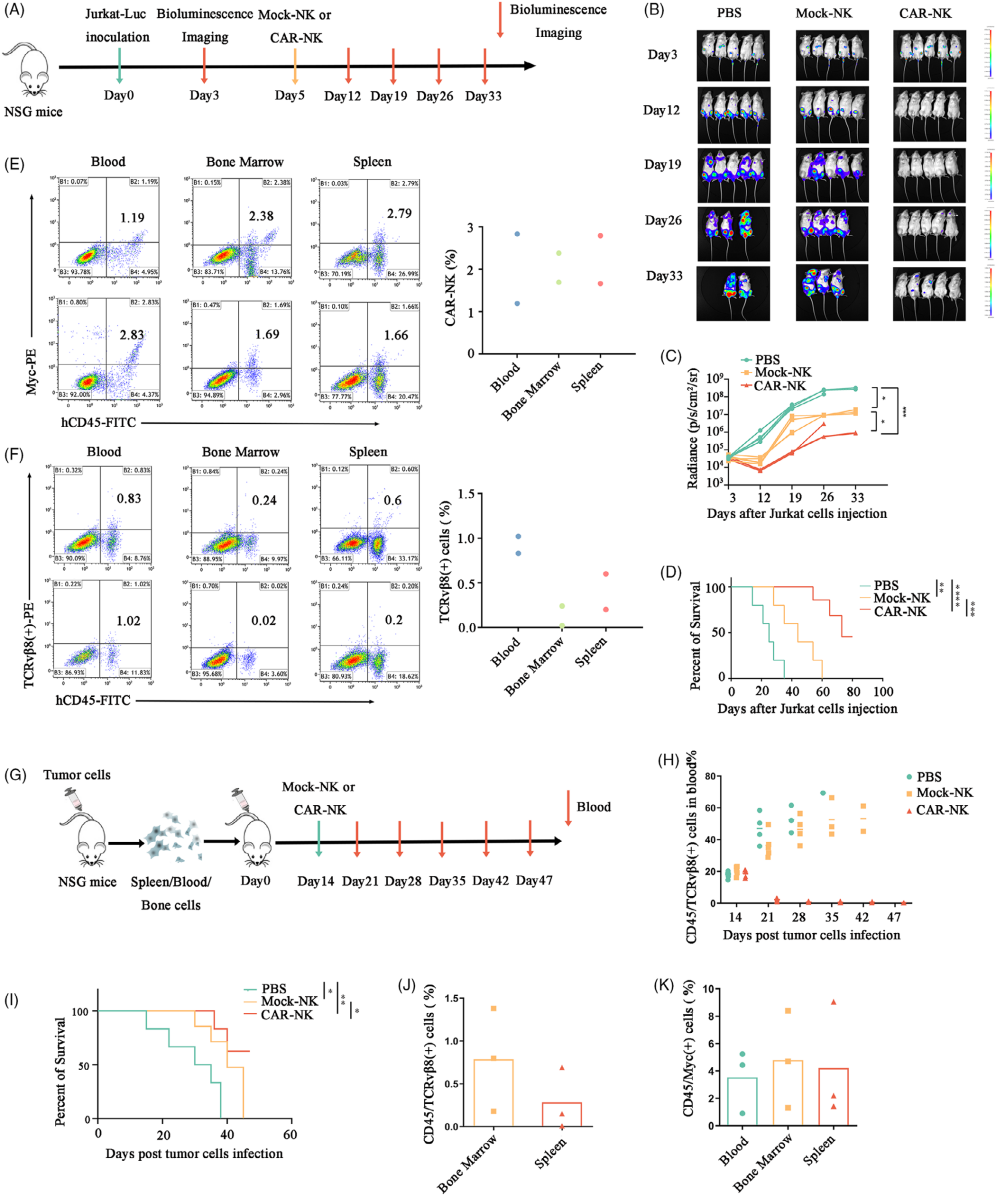
TCRvβ8^+^ chimeric antigen receptor‐natural killer (CAR‐NK) cells had anti‐tumour effects on malignant T cells in NTG mice. (A) Flowchart of CAR‐NK cell therapy. (B, C) NTG mice were treated with either 5×10^6^ CAR‐NK cells or mock NK cells after being injected with 3×10^6^ Jurkat cells for 5 days. Bioluminescence analysis was performed at specified time points after the injection of Jurkat cells to assess tumour burden. (D) The overall survival of mice after treatment with CAR‐NK, mock NK cells and PBS. (E) Analyzation of the proportions of CAR‐NK cells (hCD45^+^ /Myc^+^) in PBMC, bone marrow cells, as well as splenocytes derived from mice after CAR‐NK cell therapy on the 90th day. (F) The proportion of Jurkat (hCD45^+^ /TCRvβ8^+^) cells was also analyzed in the above samples derived from mice. (G) Flowchart of CAR‐NKs therapy. (H) NTG mice were treated with 5×10^6^ TCRvβ8 CAR‐NK or mock NK cells after being injected with 5×10^6^ T lymphoma cells for 14 days. Blood samples were taken from the orbital socket at specified times after tumour cell injection to analyze tumour burden in mice's peripheral blood. (I) The overall survival of mice after treatment with CAR‐NK, mock NK cells and PBS. (J) Analyzation of the proportions of malignant T cells (hCD45^+^ /TCRvβ8^+^) in bone marrow and spleen derived from mice after malignant T cells injection on the 47th day. (K) The proportion of CAR‐NK cells (hCD45^+^/Myc^+^) in peripheral blood, bone marrow, and spleen derived from mice.

In conclusion, we propose the development of novel CAR‐NK cells targeting TCRvβ8 malignant T‐cells. This strategy not only holds promise for eradicating T‐cell malignancies but also achieves universality and high safety, suggesting a novel therapeutic avenue for T‐cell malignancy treatment.

## AUTHOR CONTRIBUTIONS

Zhenyu Xu, Lianjun He and Lin Wan designed the studies; Lianjun He, Ye He, Yinmei He, Xing Bao, Yao Wu and Lingjie Zhou performed most of the experiments; Lin Wan, Yuqiong Yang and Ziyun Lin were responsible for collecting clinical study samples; Lianjun He, Xueyi Qian and Huimin Shao conducted data analysis; Lianjun He, Ye He, Yinmei He, Xing Bao, Lin Wan and Zhenyu Xu wrote the manuscript. All authors have approved the final manuscript.

## CONFLICT OF INTEREST STATEMENT

The authors declare no conflict of interest.

## FUNDING INFORMATION

This work was supported by the Key Project of Excellent Young Talents Support Foundation of Universities in Anhui Province (gzyqZD2021143), Project of Development of Modern Medical and Pharmaceutical Industry of Anhui Province (2021/2022), Key Projects of Natural Science Research of Universities in Anhui Province (2022AH051246), The Opening Foundation of Provincial Key Laboratory of Biological Macro‐molecules Research (LAB202201). The Youth Project of Shandong Taishan Scholars in 2024.

## ETHICS STATEMENT

All animal studies were approved by the Laboratory Animal Ethics Committee of Wannan Medical College.

## Supporting information

Supporting Information

## Data Availability

The data that support the findings of this study are available from the corresponding author upon reasonable request
