# Supplementary material for: TCRvβ8 chimeric antigen receptor natural killer cells exhibit potent preclinical activity against T‐cell malignancies
Source: Clin Transl Med. 2024 Aug 23;14(8):e70004. doi: 10.1002/ctm2.70004 (PMC11343688; doi:10.1002/ctm2.70004)
Supplement: Supplementary file 1 — Supporting Information [file CTM2-14-e70004-s001.docx]

**Supplemental methods and materials**

**Methods and materials**

**Construction of an anti-TCRvβ8 CAR lentiviral system**

The plasmid was synthesized by Nanjing GenScript Biotechnology Co., Ltd. (Nanjing, China). A TCRvβ8 specific heavy chain antibody variable fragment, selected from a humanized antibody library through phage display technology, was used to constructe the specific TCRvβ8 CAR. TCRvβ8 CAR was composed of a CD8 hinge region and a CD28 transmembrane domain, along with either 41BB or CD28 for intracellular costimulatory signaling and activating receptor CD3ζ or DAP12 molecules [1,2]. Four CAR structures, as shown in Figure 1A, were constructed. To detection and sorting of TCRvβ8 CAR-NKs conveniently in later study, a Myc tag and IRES2 promoter were inserted in tandem. The synthesized genes were cloned and inserted into the expression vector, which was the third-generation viral packaging system for BaEV-pseudotyped lentivirus.

**Production of BaEV-pseudotyped lentivirus**

3.5 × 10^6^ HEK293F cells/ml was seeded in a 125-mL flask with final volume to 30 ml. For transfection 24h prior to transfection, Dilute the cells to a final density of 4.7 × 10^6^ viable cells/mL with fresh LV-MAX™ (ThermoFisher) Production Medium.

To produce lentiviral particles, a ratio of 3:1:1:3 for the transfer vector: gag/pol: Rev: BaEV was utilized. Supernatants containing the viral particles were harvested 48h post-transfection, following by centrifugation of the cells at 1,300 × *g* in a swinging bucket centrifuge for 15 minutes. Then, the supernatant was transferred to a fresh tube and cell debris was further removed by filtration through a 0.45 μm low protein binding filter. The viral supernatant was subsequently concentrated using ultracentrifugation.

**TCRvβ8 CAR-NKs Preparation**

The primary NK cells used in this study were collected from cord blood of healthy volunteer. Firstly, PBMCs were isolated from cord blood using Ficoll and induced into NK cells using an IL-21 NK cell amplification system (Zhongying Biomedical). IL-2 (200 U/mL) and 1% penicillin/streptomycin were supplemented to the culture medium, and 5% autologous plasma was supplemented to KBM581 medium to prepare NK complete medium. According to the manufacturer’s instructions, on day 0, 1.0×10^6^/ml PBMCs were incubated with nourishing layer cells in a well plate, and the feeder cell was added at day0 and day7. Subsequently, on the third day, all the cells were collected, centrifuged and counted for the later gene transduction.

For BaEV lentiviral transduction, NK cells were resuspended at concentration of 1 × 10^6^ cells/200 µL/well in KBM581 medium supplemented with IL-2. A mixture (300 µl) of Vectofusin-1 and lentivirus in complete medium was added to 200 µL cell suspension for transduction in one well of a 24-well plate [3]. The final concentration of Vectofusin-1 was 10 µg/mL, and the final MOI was 10 for the BaEV-pseudotyped lentivirus. After centrifugation at 1500 × *g* and 32 °C for 1.5 h, the cells were cultured with lentivirus for 24 h at 37 °C with 5% CO_2_ and then they were transferred to NK cell-amplifying medium for proliferation. Then, we continued to culture NK cells for 72 h after transduction and the proportion of CAR-positive cells was evaluated using an anti-Myc antibody.

**Detection of NK cell markers**

To identify there is any differences in phenotype between Mock-NK and CAR-NK, Mock-NKs and CAR-NKs were harvested and stained with CD56-FITC, CD16-PEcy7, KIR2D-PE, KIR3D-PE, NKG2A-PE, NKG2D-PE, NKP30-PE, NKP44-PE, NKP46-PE, at 14th day after lentiviral infection. And to further identify the phenotype of CAR-NKs after stimulated with target cells. CAR-NKs were co-cultured with Jurkat cells at a ratio of 0.5:1 (CAR-NKs: Jurkat) for 24 h, and then stained with the antibodies as described above. The stained cells were analyzed by flow cytometry, and the data were analyzed with Kaluza software.

**CD107a degranulation and intracellular cytokine production**

Mock-NK and CAR-NK were co-cultured with with Jurkat cells at a ratio of 0.5:1 (CAR-NKs: Jurkat) for 8 h. Then the cells were stained with CD56-FITC, CD107a- PEcy7, and intracellularly with INFγ-PE. The stained cells were analyzed by flow cytometry, and the data were analyzed with Kaluza software.

**Cytotoxicity of CAR-NKs to cell lines in vitro**

Endogenous TCRvβ8^+^ Jurkat cells and engineered TCRvβ8^+^ CCRF-CEM cells by lentiviral, or TCRvβ8^-^ cell lines CCER-CEM, Jurkat, Nalm-6 were transduced to express firefly luciferase. For luciferase-based cytotoxicity assay, the effector(E) and target(T) cells were co-cultured at indicated E/T ratios in black 96-well flat plates (WHB) with 5 × 10^4^ target cells in a total volume of 100 μl per well in RPMI1640 medium. The supernatant from each well was collected f for cytokines analysis and 100 μL luciferase substrate (APExBIO) was added to each well to achieve a working solution concentration of 0.15mg/ml after 8 h. Bioluminescence readings were taken using a microplate reader. Cytotoxicity was calculated using the following equation, 100🞨(1-experimental condition/average control condition (targeted cells alone)).

**Cytotoxicity of CAR-NKs to patient malignant cells in vitro**

The phenotype of malignant T cells from patients was identified using the Beckman Coulter IO Test Beta Mark kit. Then, 1.0🞨10^6^ patient cells were co-cultured with CAR-NKs or Mock-NKs at a 0.5:1 E:T ratio in X-VIVO 15 (Lonza) mediums in a 24-well plate with a total volume of 500μl per well. Cells were cultured in RPMI1640 and incubated at 37C and 5% CO2. After 8 h, supernatant was collected from for cytokines, and cells were prepared for flow cytometry. The malignant cells were identified by anti-Human CD3-APC (Biolegend) and anti-Human TCRvβ8-PE (Biolegend).

**Repetitive-stimulation assay**

To mimic the long-term exposure of CAR-T cells in a tumor-bearing host in vitro, Jurkat cells were treated with mitomycin. CAR-NKs resuspended in KBM581 complete medium were stimulated with Jurkat cells at a ratio of 1:5 (NK cells: Jurkat cells). The first stimulation occurred on day 7 post-viral transduction, and NK cells were subsequently stimulated once every 7 days. On Day 4 after each round of stimulation, the CAR-T cells were used for amplification, cytotoxicity assays and T cell-subtype analysis.

To mimic the long-term exposure of CAR-T cells in a tumor-bearing host in vivo, NTG mice were engrafted IV with 3 × 10^6^ Jurkat cells. 5 days later, 5 × 10^6^ TCRvβ8 CAR-NKs cultured for 7 days after transduction and without magnetic bead sorting were injected intravenously. We collected the peripheral blood of mice once a week after CAR-NK transplantation, and the multiplication kinetics of CAR-NKs cells in vivo was monitored by the proportion of hCD45^+^/CAR^+^ cells.

**In vivo antitumor activity**

Female NOD-Prkdc^scid^ IL2rγ^tm1^ (NTG) mice, aged 6–8 weeks old and weighing 20–22 g were purchased from the SPF (Beijing) Biotechnology Co., Ltd. All animal experiments were performed following the guidelines for the Care and Use of Laboratory Animals (Ministry of Health, China, 1998) and the protocol approved by the Laboratory Animal Ethics Committee of Wannan Medical College.

NTG mice were engrafted IV with 3 × 10^6^ firefly luciferase Jurkat cells. 5 days later, either 5 × 10^6^ Mock-NKs or TCRvβ8 CAR-NKs cultured for 14 days post- transduction and without magnetic bead sorting, were injected intravenously. The tumor burden was monitored by an IVIS Lumina XR Real Time Bioluminescence Imaging System (PerkinElmer). Furthermore, bone marrow, peripheral blood, and spleen were harvested from mice 90 days after treatment with CAR-NKs. Then, single-cell suspensions were prepared for flow cytometric analysis. The spleens were manually homogenized in PBS and filtered through a 70 μm cell strainer. bone marrow cellswere obtained by flushing the femurs and tibias with PBS using a 1 ml syringe. ACK lysis buffer (BD Biosciences) was used to lyse red blood cells in all samples. Then, single-cell suspensions were labeled with monoclonal antibodies, where CAR-NKs were identified with anti-Human CD45-FITC (Biolegend) and anti Myc-PE (CST), and Jurkat cells were identified with human anti-Human CD45-FITC and anti-Human TCRvβ8-PE.

**PDX model**

To establish patient-derived xenograft (PDX) model, primary malignant T-cells

which were previously identified, were collected and frozen. A total of 5×10^6^ sample cells were injected intravenously into NTG mice, and two moribund mice were euthanized. The malignant spleen T-cells were collected after a red cell lysis procedure. Then, 5×10^6^ T-cell lymphoma cells were transplanted into 15 mice once again at the same manner [4]. After 14 days, drugs were administered following the previously described protocol. The tumor burden in the peripheral blood of mice was detected by flow cytometry every 5-7 days. On day 47, the mice were sacrificed, and tumor cells and CAR-NKs were detected by flow cytometry as previously conducted.

**Identify whether TCRvβ8 CAR-NKs affects the TCRvβ distribution in normal T-cells**

To evaluate the impact of CAR-NKs on normal T-cells, peripheral PBMCs derived from healthy adults were incubated with TCRvβ8 CAR-NKs or Mock-NKs at an effector target ratio of 0.5:1 for 24 h. Subsequently, the percentages of TCRαβ cells were analyzed by flow cytometry. Moreover, the co-cultured specimen was submitted to a commercial entity for the detection of 24 members in the TCRvβ family by flow cytometry analysis and TCR sequencing.

**Flow Cytometry**

It was used that the following fluorescently labeled monoclonal antibodies, including anti-human CD3-FITC (300406, Biolegend), anti-human TCRαβ-APC (306717, Biolegend), anti-human TCRvβ8-PE (348104, Biolegend), anti-human CD45-FITC (304006, Biolegend), anti-human CD56-PE (318306, Biolegend), anti-human CD16-PE/Cyanine7 (302016, Biolegend), as well as anti Myc-PE (3739S, CST). The Kaluza software was used to analyze the cytometry data.

**TCR RNA sequencing analyses**

For TCR sequencing, total RNA was extracted from samples using TRIzol (Invitrogen™) following the methods of Chomczynski et al. RNA quality was detected by examining the A260/A280 ratio with a Nanodrop TMOne spectrophotometer (Thermo Scientific). Approximately 2 μg of total RNA from each sample was used for TCR sequencing library preparation using the KC-DigitalTM Stranded TCR-seq Library Prep Kit for Illumina® 150 (Seqhealth Technology Co., Ltd., Wuhan, China) following the manufacturer’s instructions. The library products corresponding to 250-500 bp were enriched, quantified and finally sequenced on NovaSeq (Illumina®). Clean reads were first clustered according to the UMI sequences, in which reads with the same UMI sequence were grouped into the same cluster. Reads in the same cluster were compared to each other by pairwise alignment, and then reads with sequence identity over 95% were extracted to a new subcluster. After all subclusters were generated, multiple sequence alignment was performed to obtain one consensus sequence for each subcluster. The deduplicated consensus sequences were used for TCR-seq analysis. They were mapped to the international ImMunoGeneTics (IMGT) database by Lefrancet al. using MiXCR software (version 3.0.3) to obtain V, D and J fragment, rearrangement and CDR3 sequences.

**Single-cell RNA-sequencing analysis**

TCRvβ8 CAR-NKs were sorted by microbeads prior to the analyses. To assess the efficacy of CAR interaction with target cells that could be attributed to specific subsets of CAR-NK cells, CAR-NKs or Mock-NKs were then cocultured with Jurkat target cells at a 1:2 ratio respectively for 24 h before being harvested for library preparation sequencing. CAR-NKs or Mock-NKs were sorted by CD56 microbeads. Single-cell RNA sequencing was performed using the 10X Genomics Chromium system (Chromium Single Cell 3’ Reagent Kit, v3 chemistry) following the manufacturer’s instructions. The sorted labeled Gel Beads, samples and premixed reagents and oil were individually loaded onto incoming channels, and a two-crossed system was formed through a microflow channel network, resulting in the formation of single-cell microreactor systems (GEMs) encapsulated in oil droplets. Cells were lysed in GEMs, tagged with barcode, reverse transcribed, and cDNA was amplified. Sequencing libraries were constructed, and cDNA sequences were sequenced on-premium for quality-checking.

A principal component analysis was conducted and used the first 30 principal components were utilized for tSNE clustering. CAR-NKs and Mock-NKs were clustered separately to demonstrate the difference in subpopulations, with clusters being matched based on the correlation similarities of global gene expression. To investigate the CAR- NKs response to tumor cells, differential expression analyses were performed to identify the genes significantly upregulated in each cluster compared with all other cells by setting the log2-fold-change to ≥ 0.2 and a P value of < 0.05 from CAR-NKs or Mock-NKs. For gene sets representing specific cellular functions or pathways, we performed functional enrichment analysis for biological processes using GO with the online tool DAVID.

**Reactivity against common pathogens**

TCRvβ8^+^ T cells in healthy donor’s Peripheral T cells was deleted by magnetic bead sorting. An unsorted population was retained as control. 1🞨10^6^ T cells were co-cultured with stimulated DCs from a same donor along with peptides for cytomegalovirus (CMV), Epstein-Barr virus (EBV), or influenza (Flu) at a concentration of 1μg/ml or 10μg/ml [5]. Plates were then incubated for 24 h at 37 °C, and then supernatants were harvested and IFN-γ production was assessed by ELISA.

**Investigate the impact of antigen density on CAR functionality**

CCRF-CEM cells was overexpressed TCRvβ8 by lentiviral. The cells was then labeled with an anti-TCRvβ8 antibody, and FACS sorting was performed to isolate clones expressing varying levels of TCRvβ8. The number of molecules of TCRvβ8 antigen was estimated by the BD Quantibrite^TM^ Kit per the manufacturer’s protocol. Then, CAR-NK cells were co-cultured with these clones at a ratio of 0.2:1 or 0.5:1 for 8 h for cytotoxicity assay.

**Statistical analysis**

Student’s t test or one-way ANOVA was performed by GraphPad Prism version 8.0 (GraphPad Software Inc.). ns, *P* > 0.05; *, *P* < 0.05; **, *P* < 0.01, ***, *P* < 0.001 and ****, *P* < 0.0001.

**References**

[1] Ng YY, Tay JCK, Li Z, et al. T Cells Expressing NKG2D CAR with a DAP12 Signaling Domain Stimulate Lower Cytokine Production While Effective in Tumor Eradication. Mol Ther. 2021;29(1):75-85.

[2] Xiao L, Cen D, Gan H, et al. Adoptive Transfer of NKG2D CAR mRNA-Engineered Natural Killer Cells in Colorectal Cancer Patients. Mol Ther. 2019;27(6):1114-1125.

[3] Albinger N, Pfeifer R, Nitsche M, et al. Primary CD33-targeting CAR-NK cells for the treatment of acute myeloid leukemia. Blood Cancer J. 2022;12(4):61.

[4] Yu Y, Li J, Zhu X, et al. Humanized CD7 nanobody-based immunotoxins exhibit promising anti-T-cell acute lymphoblastic leukemia potential. Int J Nanomedicine. 2017; 12: 1969-1983.

[5] Doisne JM, Urrutia A, Lacabaratz-Porret C, et al. CD8+ T cells specific for EBV, cytomegalovirus, and influenza virus are activated during primary HIV infection. J Immunol. 2004;173(4):2410-2418.**Supplementary figures and legends**

**Figure S1**

**
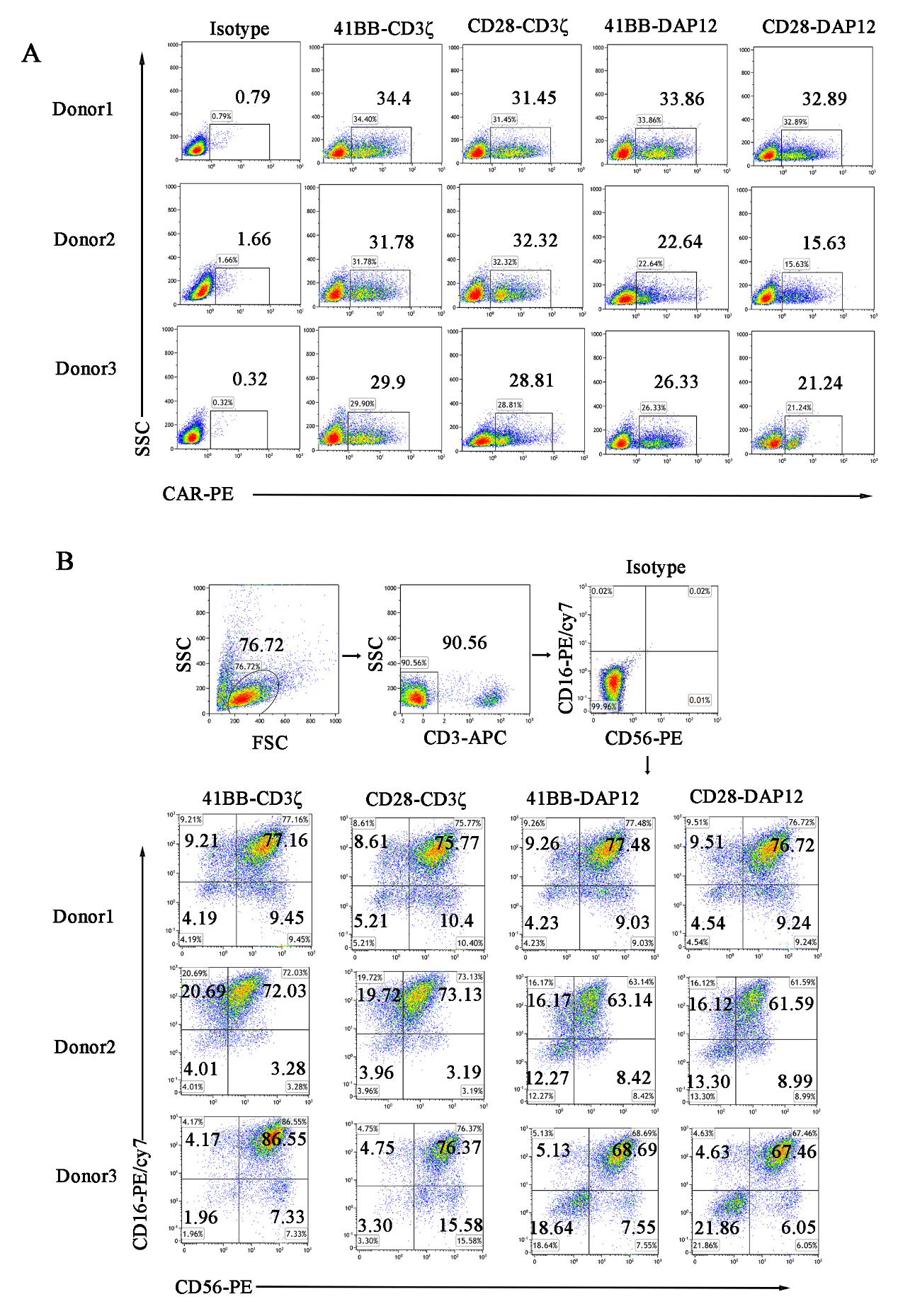
**

Figure S1. (A) The expression of TCRvβ8 CAR in 3 donors after 3 days of lentiviral infection, as detected by flow cytometry analysis. (B) Surface markers CD3, CD56 and CD16 were used for NK cell phenotyping of the CAR-NK cells from the 3 donors after 14 days of lentiviral infection.

**Figure S2**

**
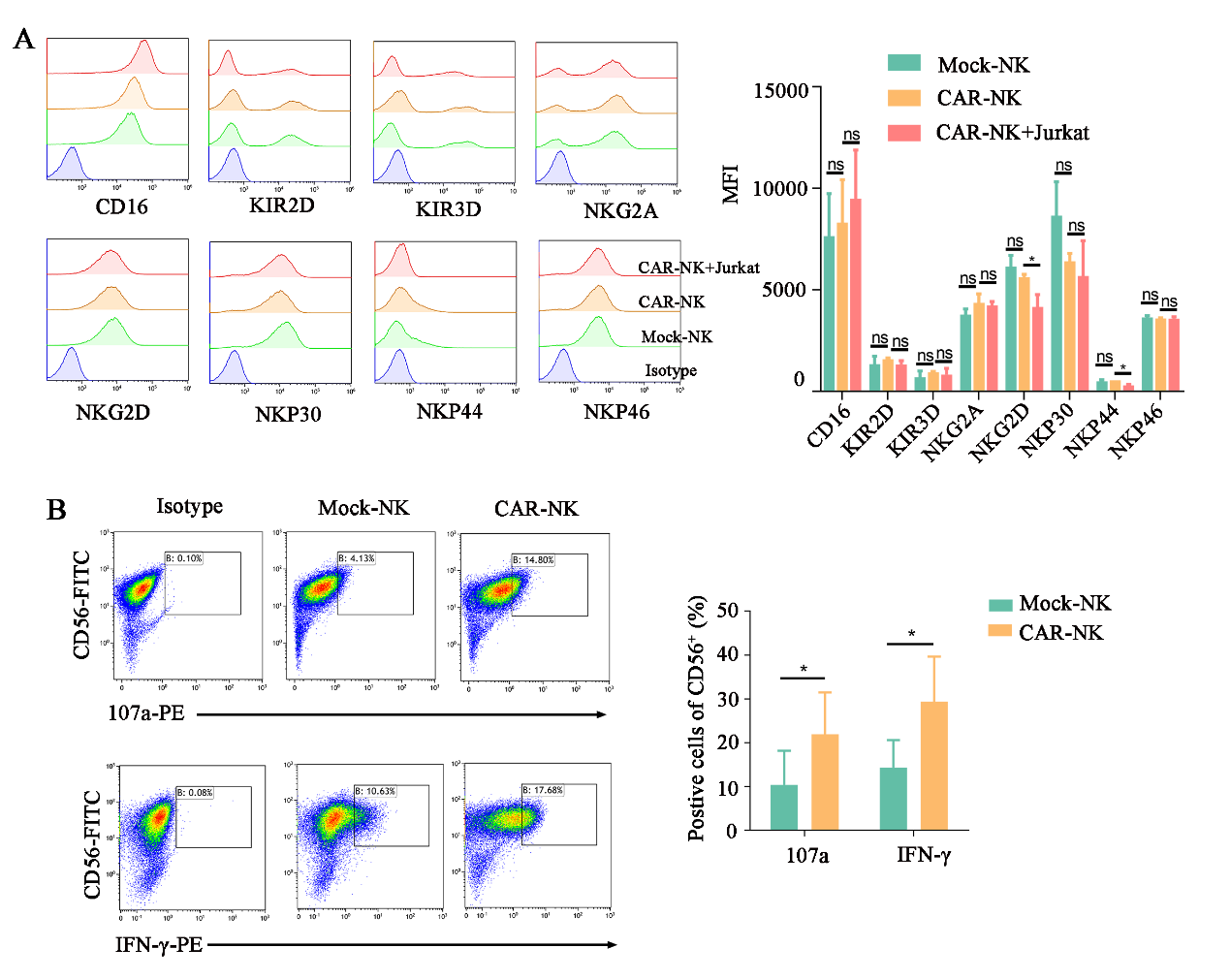
**Figure S2 The TCRvβ8 CAR vector increases NK cell functional markers. (A) The phenotypic analysis Mock-NK and CAR-NK cells with or without Jurkat cells were determined by flow cytometry analysis from 3 donors. (A) The expression of CD107a and IFN-γwas measured on CAR-NKs and Mock-NKs after co-cultured with Jurkat at an E:T ratio of 0.5:1 for 8h (n=3, mean ± s.d.).

**Figure S3**


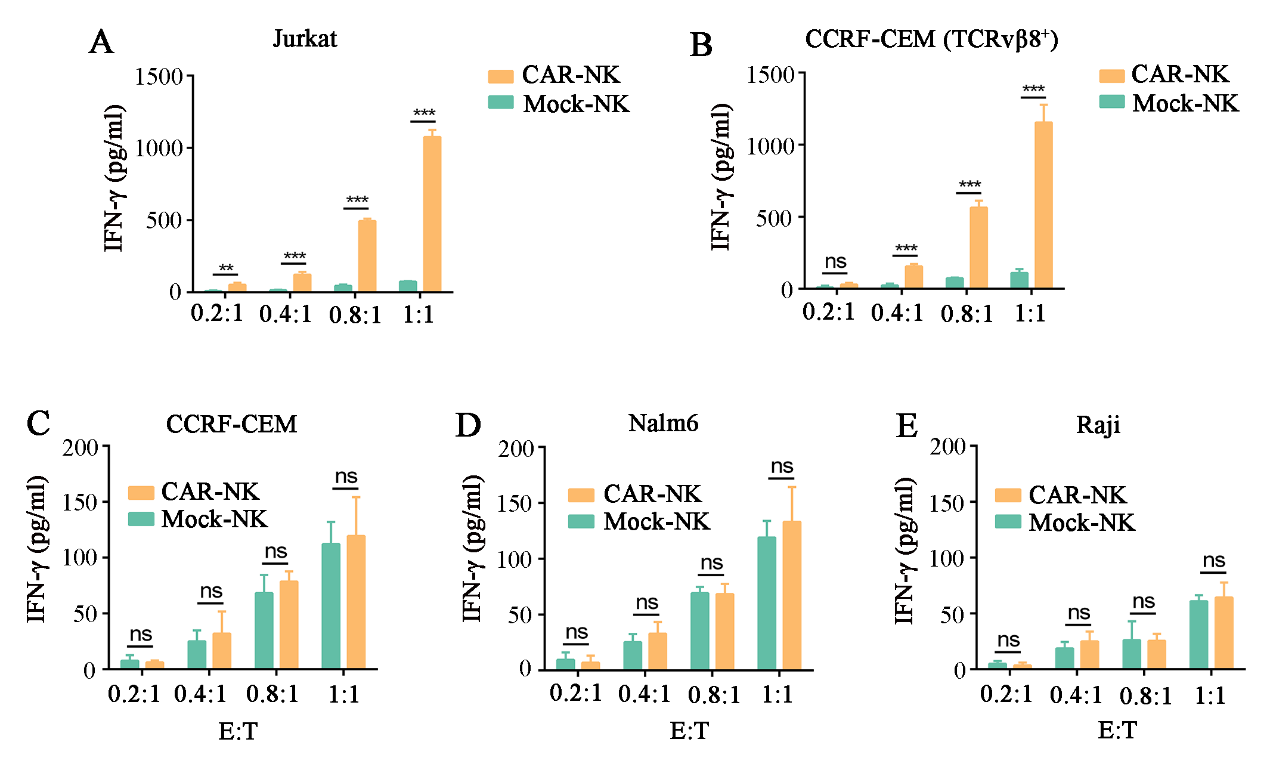


Figure S3.The expression level of IFN-γ in the supernatant of CAR-NKs or Mock-NKs after cocultured with different TCRVβ8^+^ or TCRVβ8^-^ cells at various effector-to-target ratios (n=3, mean ± s.d.). (A) Jurkat cells, (B) CCRF-CEM cells expressed exgenous TCRvβ8, (C) CCRF, (D) Nalm6, (E) Raji.

**Figure S4**


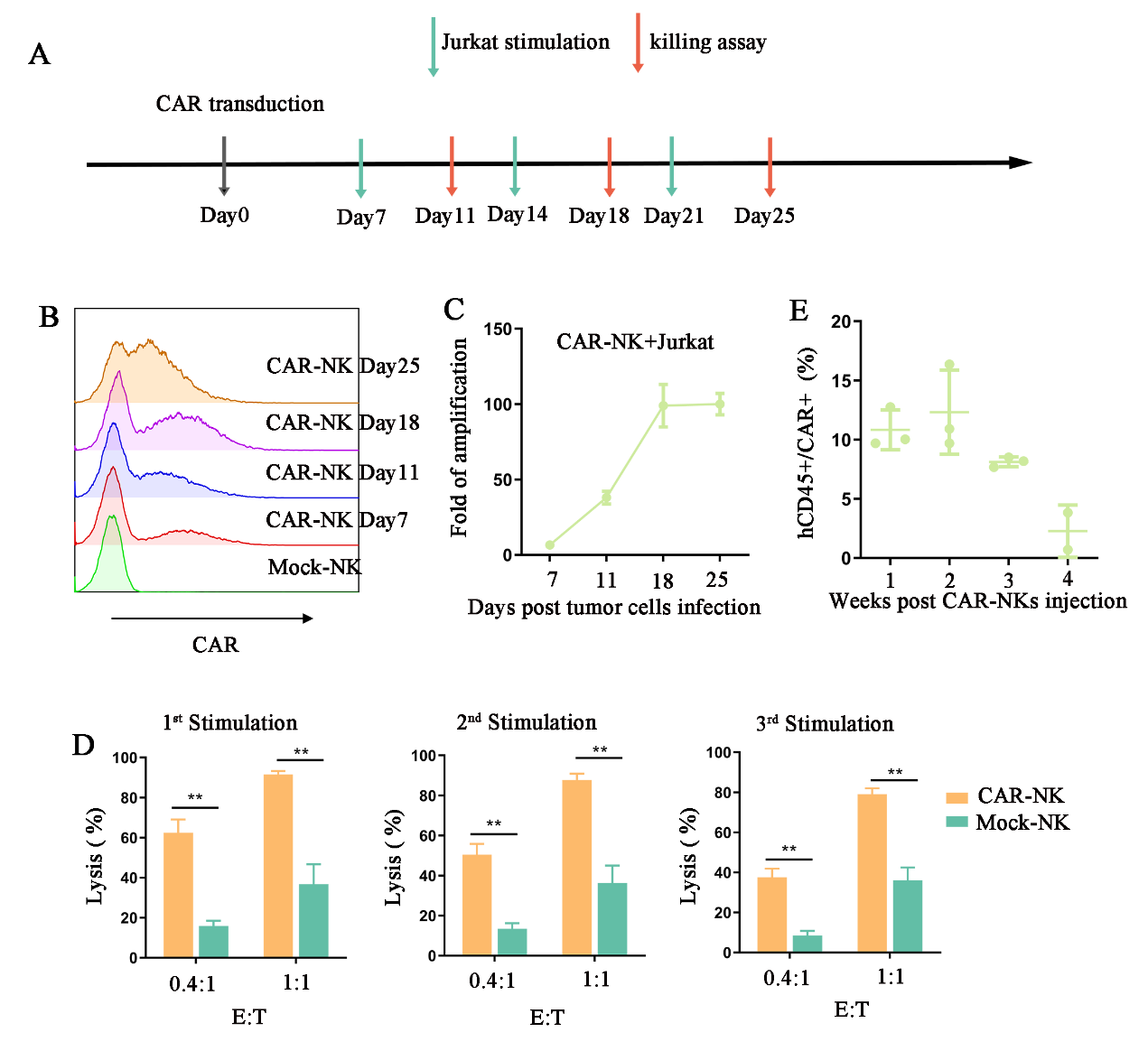


Figure S4. | Characterization of TCRvβ8 CAR-NKs after repetitive antigen exposure. (A)Schematic representation of the CAR-NKs characterization assay after long-term repetitive antigen stimulation. (B) Evaluation of CAR expression on CAR-NKs at the indicated time points by flow cytometry. (C) Growth curves of CAR-NKs over extended culture periods (n=3, mean ± s.d.). (D) CAR-NKs generated as described in Panel A were cocultured with Jurkat cells for cytotoxicity assessment (n = 3 mean ± s.d.). (E) Analysis of the proportions of malignant T cells (hCD45^+^ /TCRvβ8^+^) in peripheral blood of mice (n = 3 mean ± s.d.).
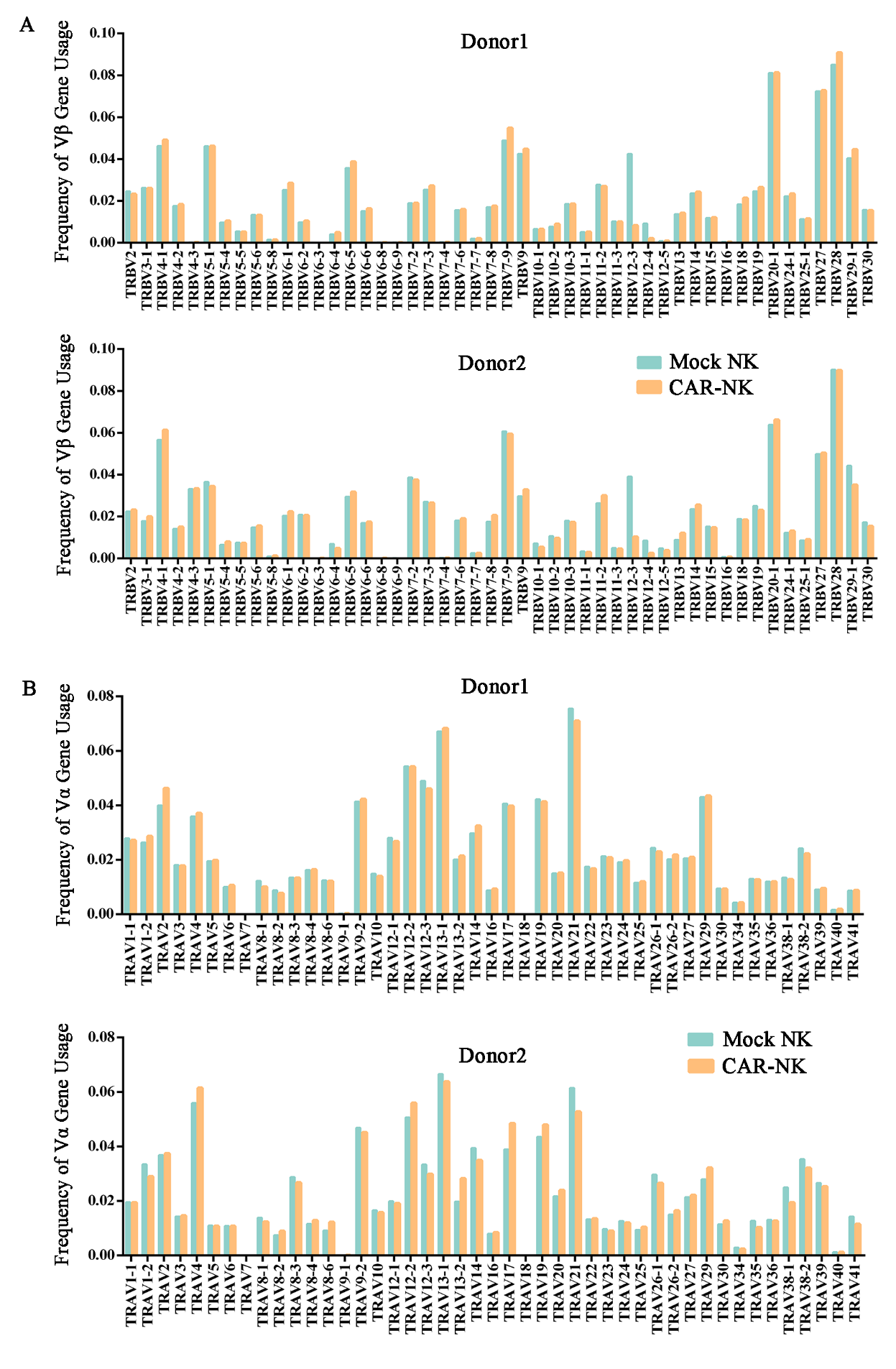


Figure S5. The proportions of (A) TCR Vβ and (B) TCR Vα genes in normal T cells from 2 donors after co-cultured with CAR-NK or mock NK cells at an effector-to-target ratio of 0.5:1 for 24 h were analyzed by TCR sequencing analysis. TCRvβ8(TRBV12-3 and TRBV12-4) was markedly decreased in TCRvβ8 CAR-NKs, but the other TCR Vβ and TCR Vα genes were not significantly changed compared to that of Mock-NKs.

**Figure S6**


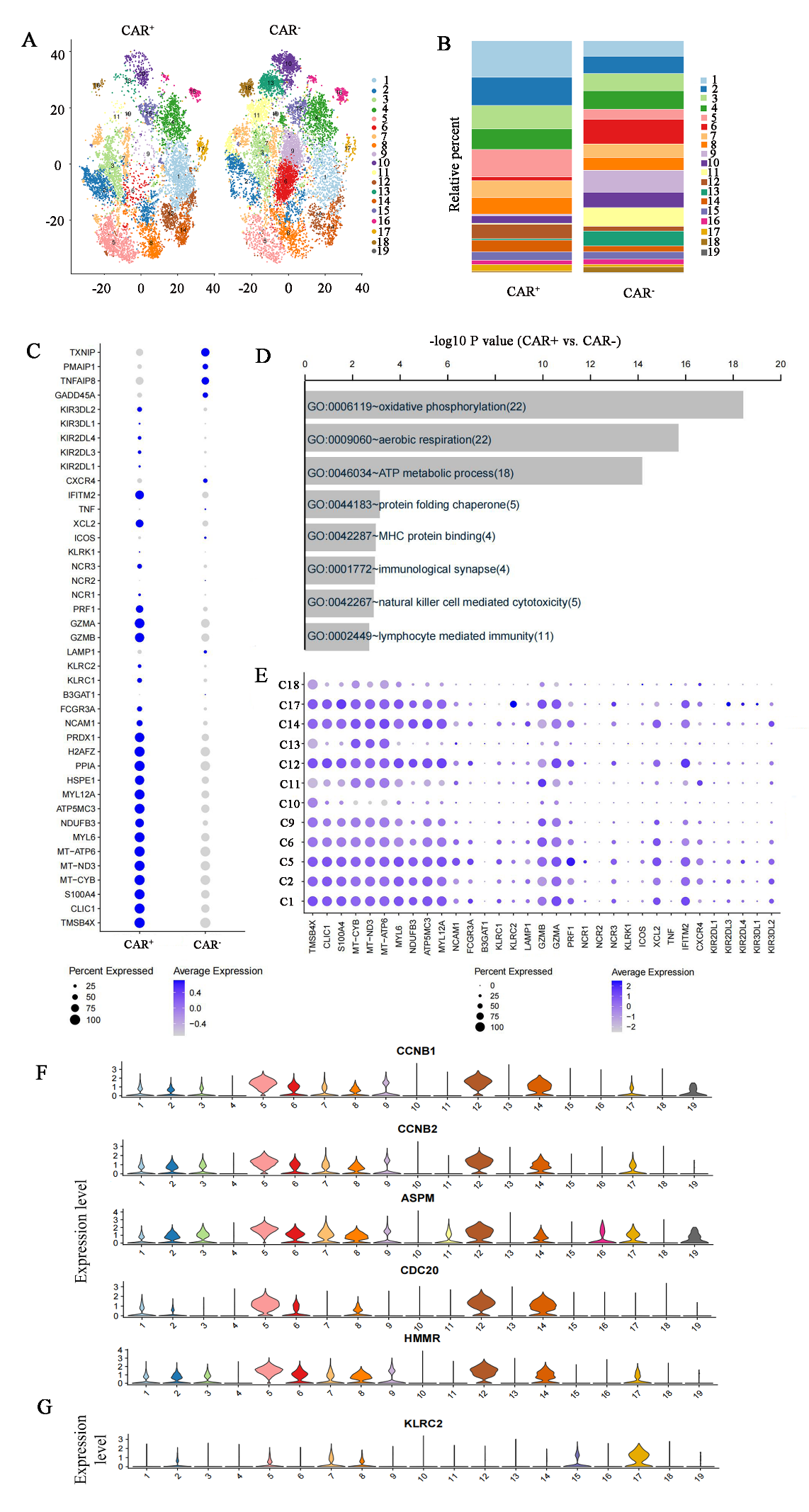


Figure S6. scRNA-seq feature analysis of TCRvβ8^+^ and TCRvβ8^-^ CAR-NK cells after co-culture with Jurkat cells. (A) t-SNE cell clustering analysis of CAR^+^ and CAR^-^ cells. (B) Percentage of cell clusters in TCRvβ8^+^ and TCRvβ8^-^ CAR-NK cells. (C) Expression dot plot of the selected gene. (D) GO enrichment analysis in CAR^+^ cells and CAR^-^ cells. (E) Expression dot plots of selected genes in Cluster.(F) Genes in key pathways involving cell cycle was found to be highly expressed in Cluster 5,12 and14, and it positively regulates T cell and B cell proliferation through iron uptake. (G) KLRC2 was found to be highly expressed in Cluster17, and it positively regulates NK cells maturation.

**Figure S7**


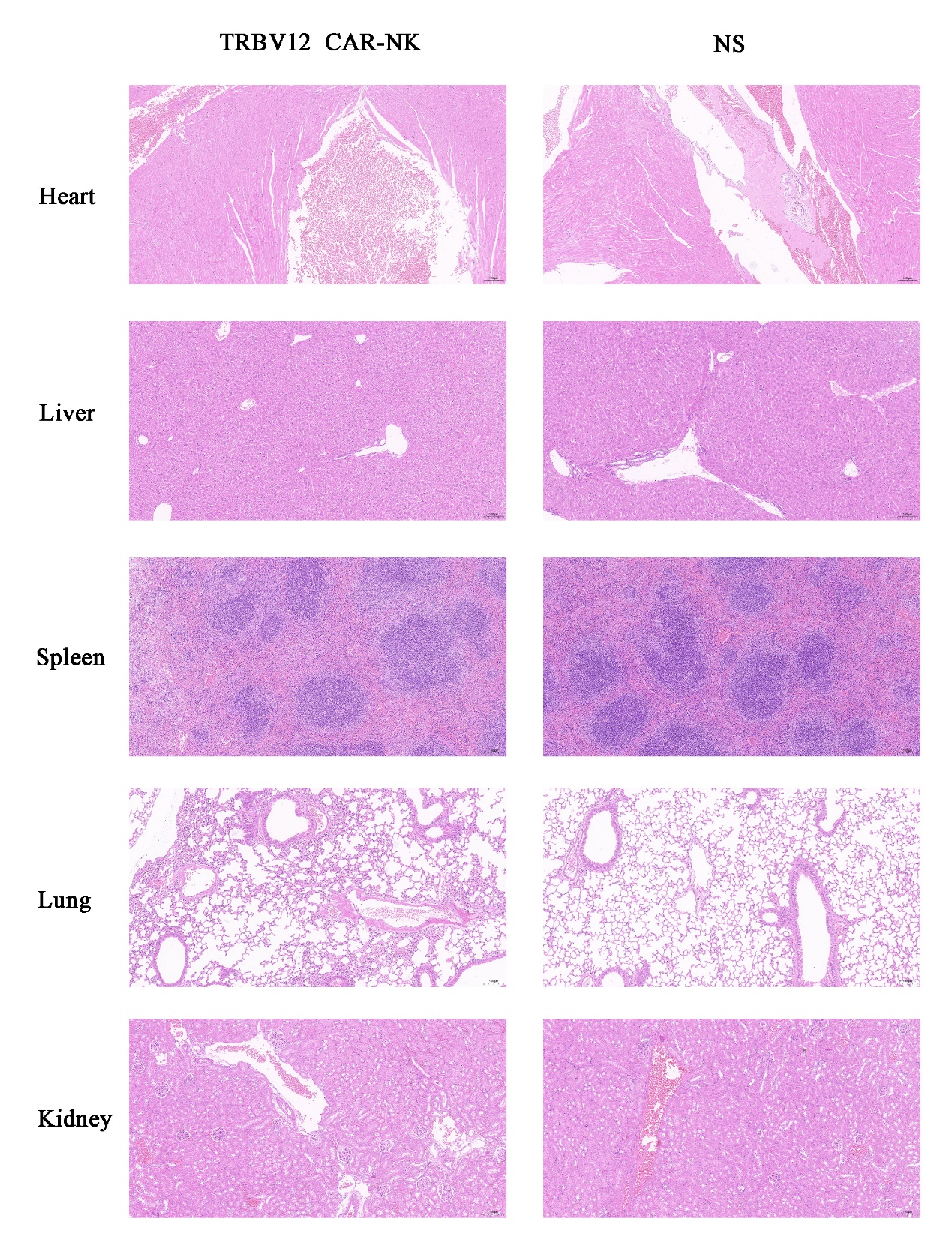


Figure S7. TCRvβ8 CAR-NKs were demonstrated tissue safety. H&E staining of the hearts, livers, spleens, lungs, and kidneys from NTG mice after treatment with TCRvβ8 CAR-NKs or saline for two weeks.
